# Collective influencers in protein interaction networks

**DOI:** 10.1038/s41598-019-40410-2

**Published:** 2019-03-08

**Authors:** T. A. Boltz, P. Devkota, Stefan Wuchty

**Affiliations:** 1 Department of Computer Science, University of Miami, Coral Gables, FL USA; 2 Department of Biology, University of Miami, Coral Gables, FL USA; 3 Sylvester Comprehensive Cancer Center, University of Miami, Miami, FL USA; 4 Center for Computational Science, University of Miami, Coral Gables, FL USA

## Abstract

Recent research increasingly shows the relevance of network based approaches for our understanding of biological systems. Analyzing human protein interaction networks, we determined collective influencers (CI), defined as network nodes that damage the integrity of the underlying networks to the utmost degree. We found that CI proteins were enriched with essential, regulatory, signaling and disease genes as well as drug targets, indicating their biological significance. Also by focusing on different organisms, we found that CI proteins had a penchant to be evolutionarily conserved as CI proteins, indicating the fundamental role that collective influencers in protein interaction networks plays for our understanding of regulation, diseases and evolution.

## Introduction

Research over the last decades increasingly shows the relevance of network based approaches for our understanding of biological systems. Generally, the importance of a protein in an interaction network is frequently considered a question of the number of interactions a given protein is involved in. Traditionally, hub genes with a large number of interactions are mostly considered important as they are enriched with essential genes^1^ and make a network more vulnerable upon their removal^2^. Such nodes frequently occupy central positions in a network as they connect different network modules. Labeled as bottlenecks^3^, such centrally placed proteins are also involved in a rising number of protein complexes^4^, suggesting that their essentiality is a consequence of their complex involvement^5,6^. Viruses and parasites target central proteins to seize control of a host cell^7,8^ while such proteins play a decisive role in different diseases^9–14^. As a corollary, proteins in a central network position can serve as a basis for the determination of disease genes, biomarkers^15–17^ and indicate points of therapeutic intervention^18,19^. However, inhibition of bottleneck nodes often compromises network integrity, and hubs frequently do not play a role in adult diseases, as such genes turn out to be embryonically lethal^11–13^. Therefore, the focus of network research shifted to the identification of influential nodes, indicating the significance of less connected proteins^20,21^. Recently, Morone and Makse^22^ introduced an optimization method to determine an optimized set of nodes termed collective influencers (CI). Such nodes were obtained via optimal percolation theory through the investigation of their propensity to damage the underlying network, strongly emphasizing the role of weakly connected nodes. Wondering if collective influencers in protein-protein interaction network carry biological significance, we expected that collective protein influencers were enriched with *e.g*. disease or essential genes. Within the human interactome, CI proteins were indeed enriched with essential, regulatory, signaling and disease genes as well as drug targets, strongly suggesting that such well-defined protein groups have significance. Furthermore, we found that CI proteins were evolutionarily conserved as CI proteins in evolutionarily conserved networks in different organisms.

## Results

**Figure 1:**
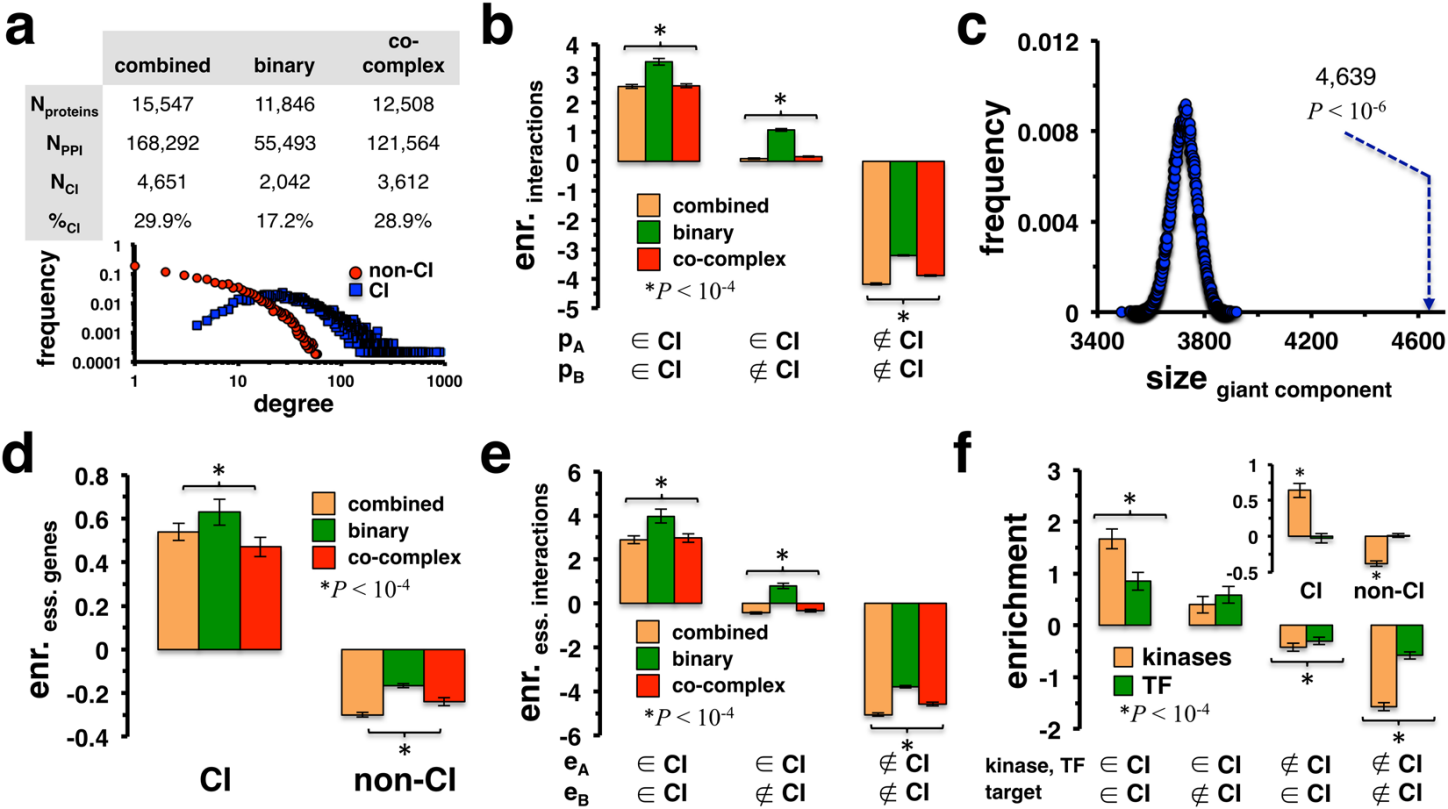
Topological and functional characteristics of collective influencers (CI) in human protein interaction networks. (**a**) In the table we present statistics of human protein interaction networks and their corresponding CIs. In particular, we accounted for binary and co-complex interactions as well as a combined interaction data set. Notably, the frequency distribution of the degrees of (non-)CI proteins in the combined network indicated that CI proteins were involved in a higher number of interactions. Furthermore, we observed that CI proteins were also frequently low-degree proteins. (**b**) Randomizing the set of CIs, we determined the enrichment of interactions between (non-)CI proteins. We found that interactions between CI proteins were enriched and appeared depleted between non-CI proteins in all networks. **(c)** CI proteins in the combined network composed a giant connected component with 4,639 CI proteins (dashed line). Such a result was significant when we randomized sets of CI proteins and determined the distribution of the sizes of the giant components thus obtained (P < 10^−6^). In (**d**), we randomized essential human proteins and observed that CIs were strongly enriched with essential genes in all networks. (**e**) Considering interactions between essential genes, we found that such interactions preferably appeared between CI proteins. (**f**) While CIs were strongly enriched with kinases, we found no significant enrichment of CIs with transcription factors in the combined network (inset). Randomizing sets of CI proteins in the combined network, we found that links between transcription factors and their corresponding targets were enriched when transcription factors were CIs. We obtained a similar result using kinase-substrate interactions.

In a protein interaction network, a set of collective influencers (CI) is defined as the minimum set of nodes that, upon deletion, destroy the largest connected component of the underlying network^22^. We determined such collective influencers in human protein-protein interaction networks as of the HINT database^23^ by applying a recursive algorithm (see Materials and Methods). Calculating a score for each protein that reflects its propensity to damage the underlying largest connected component, proteins with the largest score were removed in each step. The procedure stopped when the largest connected component disappeared, providing a list of removed nodes as collective influencers (CI). While we considered them in combination, we separately accounted for binary and co-complex interactions as well (see Materials and Methods). The table in Fig. 1a indicates that the corresponding CI sets of human interaction networks involved less than 30% of all proteins. In Supplementary Fig. 1, we observed that CI proteins on average had a higher mean degree than their non-CI counterparts in all networks. On a more fine-grained level, Fig. 1a also indicates that the degree distribution of CI proteins in the combined network featured a significantly large number of less connected nodes, a result that holds for the remaining networks as well (Supplementary Fig. 2). Furthermore, we defined a set of the 20% of proteins with the highest betweenness centrality as bottleneck nodes. Randomizing such sets, we observed that bottleneck nodes appeared enriched among CI proteins in all networks (Supplementary Fig. 3). To gain an insight into the placement of CI proteins we counted the number of interactions between pairs of (non-)CI proteins. Randomizing the set of CI proteins, we observed that CIs preferably interacted with each other, while we found the opposite when we considered non-CI proteins in all networks (Fig. 1b). Such an observation suggests that CI proteins may form a large connected component. Randomizing the set of 4,651 CI proteins in the combined network, we found that the largest connected component was significantly composed of 4,639 (99.7%) proteins (P < 10^−6^, Fig. 1c). Such results were corroborated when we considered giant components in the binary and co-complex networks (Supplementary Fig. 4).

To indicate biological significance of CI proteins in a human interaction network we hypothesized that such sets may be significantly enriched with proteins that govern biological functions. In Fig. 1d, we randomized a set of 2,708 human essential genes from the online gene essentiality database (OGEE)^24^ and the Database of Essential genes (DEG)^25^ and observed that CIs were strongly enriched with essential genes in all networks. Previously, essential genes were found to form large connected components in protein interaction networks^26^, prompting us to hypothesize that interactions between essential genes may be driven by CI proteins. Indeed, we observed that interactions between essential genes were enriched between CI proteins when we randomized sets of essential genes in all networks (Fig. 1e).

Assuming that CI proteins may significantly contribute to control processes, we hypothesized that transcription factors may appear appreciably in CI sets. Furthermore, we surmised that the same logic applies to kinases. Utilizing a set of 501 kinases from the Kinome NetworkX database^27^, we observed that kinases were strongly enriched in CI sets, while we found no corresponding signal when we considered 1,471 manually curated transcription factors^28,29^ in the combined network (inset, Fig. 1f). Such results were corroborated when we considered the enrichment of kinases in the CI sets of binary and co-complex networks. However, transcription factors were significantly enriched in the set of CIs in the binary network, while we found the opposite in the co-complex network (Supplementary Fig. 5). As a corollary, we hypothesized that interactions between transcription factors and their targets, as well as interactions between kinases and their substrates, may be dominated by CI proteins. Randomizing CI proteins in the combined network, we indeed found that such links were enriched when transcription factors and kinases and their targets and substrates were CIs (Fig. 1f). Such results were corroborated in the binary and co-complex network as well (Supplementary Fig. 6).

**Figure 2:**
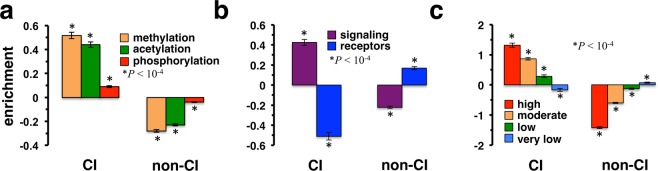
Signaling genes and translational regulation of collective influencers (CI) in human protein interaction networks. (**a**) As for recipients of post-translational modifications, we observed that CIs were strongly enriched with methylation and acetylation targets in the combined network. While still significant, CIs were less enriched with phosphorylation targets. (**b**) While signaling proteins without membrane domains appeared strongly enriched with CIs, we found the opposite when we considered receptors that carried a trans-membrane protein in the combined network. (**c**) As a measure of translational regulation, we found that CIs in the combined network were significantly enriched with high copy number proteins, while they were depleted with low copy number proteins.

Other indications of biological relevance are recipients of post-translational modifications, suggesting that such modified proteins may be enriched with collective influencers. Utilizing sets of proteins that were methylated, acetylated or phosphorylated, we found that collective influencers were generally enriched with proteins with post-translational modifications in the combined (Fig. 2a) as well as binary and co-complex networks (Supplementary Fig. 7). Furthermore, we considered proteins involved in signaling functions that have no trans-membrane domains, and such proteins appeared enriched as well in the CI set in the combined network (Fig. 2b). In turn, we observed the opposite when we considered receptor proteins with trans-membrane domains. Such results were corroborated in the binary and co-complex networks (Supplementary Fig. 8). In Fig. 2c we analyzed the role of protein steady state abundance in cell lines as a measure of translational regulation. In particular, we observed that CI proteins were strongly enriched with 810^30^ high copy number proteins in the combined network. Notably, the level of enrichments of 2,401 moderately and 1,977 low copy number proteins were significant as well but were steadily decreasing. Finally, 2,102 very low copy number proteins were depleted with CI proteins, results that held in the binary and co-complex networks as well (Supplementary Fig. 9).

**Figure 3:**
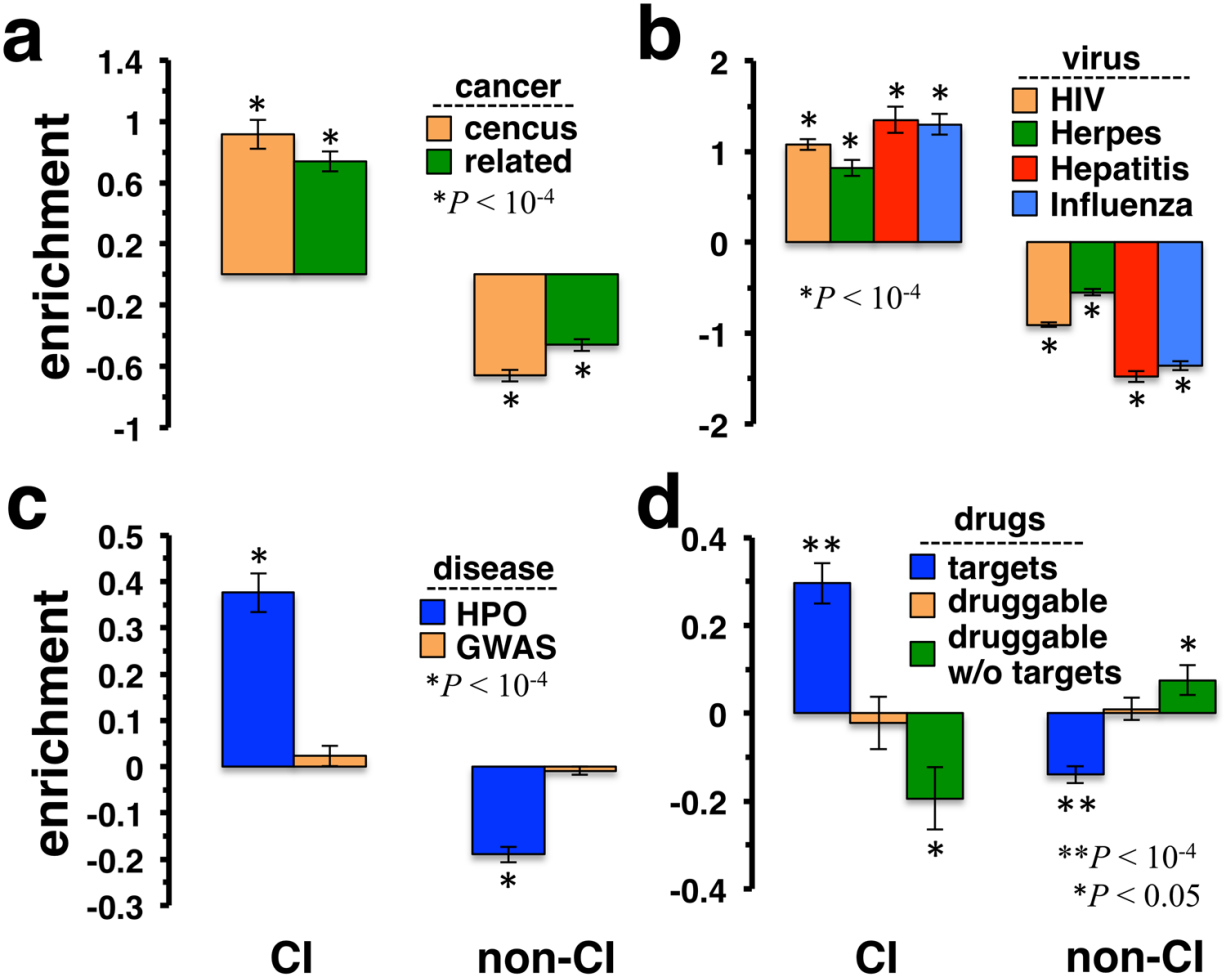
Collective influencers (CI) in the combined network were enriched with disease genes and drug targets. In **(a)** we determined the enrichment of cancer genes in sets of (non-)CI proteins. Using a compilation of census cancer genes and a set of onco- and tumor-suppressor genes, we found that such cancer genes strongly appeared enriched with CI proteins. (**b**) Similarly, we observed that various viruses preferably targeted CI proteins. **(c)** Utilizing disease gene information from genetic (HPO) sources, we found that CIs were significantly enriched with disease genes. In turn, no significant signal emerged when we considered genomic (GWAS) disease gene sources. (**d**) As a corollary, FDA approved drug targets were enriched with CIs while druggable genes appeared diluted with CIs.

Furthermore, we analyzed the role of CI proteins in diseases and drug development, assuming that CI proteins may drive the transition between healthy and disease conditions. Utilizing a set of genes that were annotated by the Sanger Center as causally implicated in oncogenesis^31^, we observed that CI proteins were enriched with such cancer genes in the combined network (Fig. 3a). To further substantiate our observations, we considered a different set of 1,259 of onco- and tumor suppressor genes^32^ that were predicted as cancer-related and obtained similar results. As for viral infections, we utilized sets of proteins that were targeted by the HIV, Hepatitis C, Herpes and Influenza virus from the HPIDB database^33^. In all cases we found that CI proteins were enriched with viral targets (Fig. 3b). Focusing on disease genes more generally, we considered a set of 2,661 genes as of the HPO database^34,35^ that carry disease-causing mutations. Specifically, such disease genes were significantly enriched in sets of CIs (Fig. 3c). Focusing on 11,002 disease genes that were identified from GWAS studies^36^ we surprisingly observed no relevant enrichments (Fig. 3c). As a corollary, we hypothesized that drug targets may preferably enriched with CI proteins as well. Investigating a set of 2,289 drug targets that were approved by the Food and Drug Administration (FDA)^37^, we observed that drug targets predominantly appeared in groups of CI proteins (Fig. 3d). Furthermore, we considered a set of 2,436 proteins deemed druggable given that they carry protein folds, which favor interactions with chemical compounds. While we did not find any enrichment signals, we observed that CI proteins appeared diluted in a subset of 1,848 druggable genes that were not approved drug targets.

**Figure 4:**
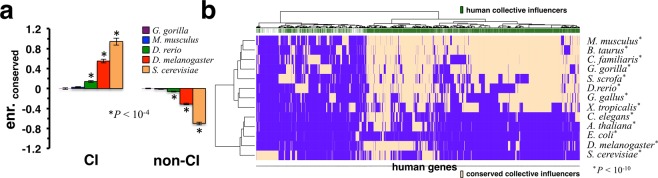
Evolutionary conservation of collective influencers (CI). In (**a**) we determined the propensity of (non-)CI proteins in the human combined network to be evolutionarily conserved in other organisms. Surprisingly, we observed that CIs were significantly more conserved in distant organisms. In (**b**) we mapped all human genes to evolutionarily conserved CIs in organism specific networks of interactions. Notably, human CIs were significantly enriched with CIs in other organisms (P < 10^−10^, Fisher’s exact test).

The observed biological relevance of CI proteins prompted us to hypothesize that the role of such proteins are conserved in evolution. In particular, we utilized orthologous groups of proteins as found in the OMA database^38^. In Fig. 4a, we determined the propensity of (non-)CI proteins in the human combined network to be evolutionarily conserved in different organisms, including mouse, gorilla, zebrafish, fruit fly and yeast, by randomly sampling sets of collective influencers. Surprisingly, we observed that CIs were increasingly conserved in more evolutionarily distant organisms such as fruit fly and yeast. Considering the propensity of interacting pairs of interacting human (non-)CI proteins, we found that interacting pairs of CI proteins were preferably conserved while we found the opposite for interacting non-CI proteins (Supplementary Fig. 10). Still, our results slightly indicate that evolutionary signals were stronger when we considered more evolutionary distant organisms. At this point our considerations only accounted for evolutionary conservation of collective influencers if they had orthologs in other organisms, ignoring any network aspects of orthologs in other organisms. Therefore, we wondered if CI proteins may be preferably conserved as CI proteins in other organisms as well. Inferring networks of protein-protein interactions in other organisms, we considered an interaction to be conserved if the interacting proteins in the human combined network had orthologs in the underlying other organism. For example, we found 61,619 conserved interactions between 8,617 mouse proteins and 2,323 CIs in the inferred mouse specific protein interaction network. In Fig. 4b, we mapped human proteins to conserved CIs that we found in different organisms. Notably, the heatmap indicates that human CIs significantly overlapped with conserved CIs in closely related organisms, results that we found significant using Fisher’s exact test (P < 10^−10^).

## Discussion

Here, we determined collective influencers (CI) in protein interaction networks that were defined as the subset of proteins that damaged the underlying network to the largest possible degree. The underlying model is based on optimal percolation of a network that also considers the influence on other nodes in the network that are a given shortest distance away from a removed node in question. As indicated in^22^ minimizing the number of removed nodes to optimally percolate the network is tractable only when nearest neighbors of removed nodes are considered. However, the optimization procedure becomes hard if nodes are considered that are further away. Generally, finding collective influencers has been classified as NP-hard^39^, prompting the application of a heuristic that is not guaranteed to find the exact solution. Furthermore, the underlying percolation model was largely based on the assumption of locally tree-like random graphs. Notably, protein-protein interaction webs do not fall into this class of networks, sugesting that the observed sets of collective influencers portray a rather rough approximation of the exact solution.

Despite these limitations, we still found that collective influencers in protein interaction networks carry enough biological weight to emphasize their biological and topological relevance. Generally, we observed that the source of the underlying network information did not play a discriminatory role when we considered topological characteristics of CI proteins. While such CI proteins were preferably highly connected, their degree distribution suggests that a fair amount of CI proteins are rather sparsely connected. Such an observation indicates that high local centrality alone is no sufficient criterion to be relevant for the integrity of the underlying network. In turn, the way to determine CIs considers the whole network, providing an optimal smallest set of strategically placed proteins. As a consequence, we also found that CI proteins were enriched with bottleneck proteins that had a high betweenness centrality, indicating a global measure of centrality. As another indicator of topological relevance, we observed that interactions preferably appeared between CI proteins, but were diluted between non-CI proteins. Such a result is probably rooted in the way CIs are determined as CIs occupy central positions that are crucial for the integrity of the network.

With such results that highlight the topological placement of CI proteins, the question remains if such characteristics translate into a governing, biological role in the underlying networks. As CI proteins are crucial for the integrity of the underlying protein interaction networks, we confirmed our obvious hypothesis that CIs may be essential genes. As a corollary of our observation that essential genes significantly set up a large connected network component, the propensity of CI proteins to interact with each other was enforced when we considered essential CI proteins. In a similar vein, the central placement of CI proteins may support functional interactions that exert biological control. While CI proteins were strongly enriched with kinases, we did not find any significant signals when we considered transcription factors. Such a result may be rooted in the fact that transcription factors are ubiquitous in terms of gene regulation, while kinases may tap their central topological position to collect and disseminate biological information. As a corollary of this hypothesis, we expected that the controlling (i.e. transcription factors, kinases) and controlled entity (i.e. target genes, substrates) were CI proteins. Indeed, we found that such interactions appeared most enriched when both transcription factors/kinases and targets/substrates were CI proteins, while we found the opposite when we considered non-CI proteins. Given that the topological placement of CI nodes was heralded as crucial for spreading information in different non-biological networks^22^, a transcription factor or kinase that is a CI may have an advantage to efficiently disseminate signals through corresponding interactions. In turn, a signal that is mediated by the expression levels of a target gene or the phosphorylation of substrates may have stronger efficacy when distributed through the interactions of a CI protein. As a consequence, CIs may be considered a complement that allows transcription and phosphorylation events to efficiently control biological processes. To corroborate this point, we also found that methylation and acetylation targets, as well as proteins that appear in signaling pathways, were enriched with CI proteins. Assuming that CI proteins play a fundamental role in the dissemination of information, we further hypothesized that such proteins need to be highly abundant. Indeed, we found that CI proteins were enriched with highly abundant proteins, a correlation that decreased with lower abundance.

In terms of network biology, mutations that cause diseases mediate their influence through the interactions of an afflicted protein^11^. As a consequence of this assumption, we hypothesized that CI proteins may be enriched with disease genes as the topological placement of CI proteins allows fast transmission of a perturbation. Indeed, we found that cancer genes, viral targets and disease genes that carry mutations were found enriched in the set of CI proteins, while they were diluted among non-CI proteins. Surprisingly, we did not find any significant results when we considered genomic disease gene sources from genome-wide association studies, most likely reflecting the fact that GWAS identify genomic regions but not specific disease causing genes^21^. As CI proteins tend to shape large connected components, such patterns resemble the propensity of disease genes to shape connected subnetworks^11,40^. Usually, topological properties such as hubs and modules are used to identify disease genes, suggesting that CI proteins may serve as a complementary framework for network medicine^21^. Furthermore, we corroborated the role of CI proteins as central to dissemination of cellular information in the transition from a disease to a healthy cellular state, when we found that drug targets were enriched with CI proteins. Surprisingly, druggable genes generally showed the opposite, suggesting that protein domain-folds that can interact with drugs alone are no good indicators of a potential drug target.

Finally, we investigated the evolutionary characteristics of CI proteins, observing that human CI proteins were enriched with orthologs in more distant organisms. Such an observation was surprising at first, as we expected that human CI proteins may be rather conserved in closely related organisms. In turn, however, conservation over long distances in the phylogenetic trees may require genes that prevailed in evolution under strong selective pressure. Such an assumption may therefore indicate that such genes may have important functional and topological roles that are reflected by their presence of collective influencers in conserved topological network patterns. By constructing conserved interolog networks in different organisms that were inferred from a human interaction network, we observed that human CI proteins are preferably conserved as CI proteins in closely related organism specific networks. Such an observation suggests that topological aspects of the underlying networks are preserved in evolution as well.

As a closing remark, the topological and biological characteristics of collective influencers are reminiscent of properties of protein sets in protein interaction network that constitute minimum dominating sets (MDSet)^20,41^. In particular, the investigation of MDSet proteins in protein interaction network emphasized the role of centrally placed and weakly connected nodes as well, suggesting similarities in the way collective influencers and MDSet proteins were determined. However, the determination of minimum dominating sets aimed at the determination of the smallest set of nodes in a network, securing that each node in the network is either participating in the MDSet or adjacent to a MDSet node. While collective influencers were selected as the smallest set of nodes to percolate a network, MDSet nodes are rather considered as topological controllers, as each node can easily be reached by nodes in the MDSet.

## Materials and Methods

### Protein-protein interactions

We utilized a total of 168,292 high quality protein interactions between 15,547 human proteins from the HINT database^23^. Furthermore, we accounted for 55,493 binary interactions between 11,846 proteins and 121,564 co-complex interactions between 12,508 proteins from the HINT database^23^.

### Determination of collective influencers

The determination of collective influencers is based on optimal percolation, aiming at the determination of a minimum set of nodes that fragments the underlying network. In the follwing we briefly introduce the model (details of the underlying model can be found in^22^).

The collective influence theory for optimal percolation is based on the message passing equations of the percolation process. For a directed link from *i* to *j* ${v}_{i\to j}$ represents the probability that node *i* belongs to the giant component *G* of a network with *N* nodes and *M* edges in the absence of node *j* that can be defined as ${v}_{i\to j}={n}_{i}[1-\prod _{k\in \partial i\backslash j}(1-{v}_{k\to i})]$. In particular, ${n}_{i}=1$ indicates that node *i* is removed and ${n}_{i}=0$ otherwise, while $\partial i\backslash j$ refers to the nearest neighbors of node *i* excluding *j*.

Such an equation can be evaluated at $\{{v}_{i\to j}=0\}$ with the aid of a $2M\times 2M$ coupling matrix defined as ${ {\mathcal M} }_{k\to l,i\to j}=\frac{\partial {v}_{i\to j}}{\partial {v}_{k\to l}}{|}_{\{{v}_{i\to j}=0\}}$. Its largest eigenvalue $\lambda ({\boldsymbol{n}};q)$ corresponds to the solution that the size of the giant component *G* = 0 (i.e. the network vanishes), where *q* is the fraction of removed nodes corresponding to vector ***n*** that indicates if node *i* is removed $({n}_{i}=1)$ or remains in the network $({n}_{i}=0)$. For locally tree-like random graphs ${\mathcal M}$ can be expressed through a non-backtracking matrix ${\mathcal B}$, ${ {\mathcal M} }_{k\to l,i\to j}={n}_{i}{ {\mathcal B} }_{k\to l,i\to j}$, where ${ {\mathcal B} }_{k\to l,i\to j}=1$, if $l=i$ and $k\ne j$, and 0 otherwise. In other words, the non-backtracking matrix only considers consecutive, directed edges, $k\to i\to j$ where node *j* can not link back to *k*.

The optimal influence problem for a given *q* can now be formulated as finding the optimal configuration ***n*** that *minimizes* the largest eigenvalue $\lambda ({\boldsymbol{n}};q)$ of ${\mathcal M}$. At a critical fraction of removed nodes ${q}_{c}$, only one configuration of the vector ${{\boldsymbol{n}}}^{\ast }$ exists such that $\lambda ({{\boldsymbol{n}}}^{\ast };{q}_{c})=1$. In practice, the largest eigenvalue can be calculated by the power method where $\lambda ({\boldsymbol{n}};q)=\mathop{\mathrm{lim}}\limits_{l\to \infty }{[\frac{|{{\boldsymbol{w}}}_{\ell }({\boldsymbol{n}})|}{|{{\boldsymbol{w}}}_{0}|}]}^{1/\ell }$where $|{{\boldsymbol{w}}}_{\ell }({\boldsymbol{n}})|$ is the $\ell$ iteration of ${\mathcal M}$ on the initial vector ${{\boldsymbol{w}}}_{0}$: $|{{\boldsymbol{w}}}_{\ell }({\boldsymbol{n}})|=|{ {\mathcal M} }^{\ell }{{\boldsymbol{w}}}_{0}|$. To find the optimal configuration of ${\boldsymbol{n}}$ $|{{\boldsymbol{w}}}_{\ell }({\boldsymbol{n}})|$ needs to be minimized for a finite $\ell$. Through a proper simplification (details in^22^), $|{{\boldsymbol{w}}}_{\ell }({\boldsymbol{n}})|\,$ is approximated as

$$|{{\boldsymbol{w}}}_{\ell }({\boldsymbol{n}}){|}^{2}=\sum _{i=1}^{N}({k}_{i}-1)\sum _{j\in \partial Ball(i,\,2\ell -1)}(\prod _{k\in {\wp }_{2\ell -1}(i,j)}{n}_{k})({k}_{j}-1),$$

where $\partial Ball(i,\,\ell )$ is the set of nodes a shortest path length $\ell$ away from node *i*, ${\wp }_{\ell }(i,j)$ is the shortest path of length $\ell$ connecting *i* and *j* and ${k}_{i}$ is the degree of node *i*. Based on this equation, an energy function for each configuration ${\boldsymbol{n}}$ can be defined as

$${E}_{\ell }({\boldsymbol{n}})=\sum _{i=1}^{N}({k}_{i}-1)\sum _{j\in \partial Ball(i,\,\ell )}(\prod _{k\in {\wp }_{\ell }(i,j)}{n}_{k})({k}_{j}-1).$$

While for $\ell =1,\,{E}_{\ell }({\boldsymbol{n}})$ can easily be optimized, the optimization procedure becomes hard for $\ell \ge 2$. Therefore, a heuristic was applied that allows the minimization of the largest eigenvalue of ${\mathcal M}$ for a given $\ell$ through a greedy algorithm, approximating ${E}_{\ell }({\boldsymbol{n}})$. In fact, ${E}_{\ell }({\boldsymbol{n}})$ can be rewritten as the sum of collective influences of single nodes ${E}_{\ell }({\boldsymbol{n}})=\sum _{i=1}^{N}CI(i)\,$, and the collective influence *CI* of a node *i* with degree *k*_*i*_ in a sphere of influence $\partial Ball(i,\,\ell )$ of size $\ell$ is defined as $C{I}_{i}=({k}_{i}-1)\sum _{j\in \partial Ball(i,\,\ell )}({k}_{j}-1)$.

The main idea of the heuristic is to remove the nodes that cause largest decrease of energy function ${E}_{\ell }({\boldsymbol{n}})$. In each step of the algorithm, the CI score for each node *i* in the largest connected compoenent of the underlying network was calculated by the above formula, where we define $\partial Ball(i,\,\ell =1)$ being the set of nodes that are connected to node *i*. After sorting proteins according to their CI score, nodes were removed with the highest score, and CI scores for all nodes in the remaining largest connected component were recalculated. The adaptive removal continued until the giant component was completely fragmented (i.e. *G(q)* = 0).

### Betweenness centrality

As a global measure of its centrality, we calculated node betweenness, indicating a node’s appearance in shortest paths through the whole network. In particular, we defined betweenness centrality *c*_*B*_ of a node *v* as ${c}_{B}=\sum _{s\ne t\ne v\in V}{\sigma }_{st}(v)/{\sigma }_{st}$, where ${\sigma }_{st}$ was the number of shortest paths between proteins *s* and *t*. Furthermore, ${\sigma }_{st}(v)$ was the number of shortest paths running through *v*. Based on this measure, we defined a set of bottleneck proteins as the top 20% of proteins with the highest betweenness.

### Functional sets of genes

We collected 2,708 human essential genes from the online gene essentiality database (OGEE)^24^ and the Database of Essential genes (DEG)^25^.

As for human transcription factors and kinases, we used a set of 1,471 manually curated sequence-specific DNA-binding transcription factors^28,29^ and 501 genes from the Kinome NetworkX database^27^ that collects kinase information from the literature and other databases. Furthermore, this database provided 7,346 interactions between 357 kinases and 2,181 substrates. We collected 95,722 links between 209 human transcription factor and 8,910 human genes from the TRANSFAC^42^ database as provided by mSigDB^43^.

As for post-translational modifications (PTMs), we used 17,511 phosphorylated proteins, 6,928 acetylated proteins and 5,418 methylated proteins from the PhosphoSitePlus database^44^.

As for signaling genes, we used 4,408 genes that were annotated with a signaling function without receptor domain function from Gene Ontology (GO)^45^ as well as 5,701 genes that carried a trans-membrane protein domain^46^.

Furthermore, we utilized a set of protein abundances^30^ in human cell lines. In particular, we accounted for 810 highly (>100,000 copies), 2,401 moderately (5000–100,000), 1,977 lowly (500–5000) and 2,102 very lowly abundant proteins (<500).

### Disease genes and drug targets

As representative sets of cancer genes, we used 568 genes that were annotated by the Sanger Center as causally implicated in oncogenesis^31^ and 1,259 onco- and tumor suppressor genes that were predicted as cancer-related^32^. As for viral infections, we obtained 988 human proteins that were targeted by the Hepatitis C virus, as well as 2,157 targets of the Herpes simplex virus, 872 targets of HIV-1 and 2,358 targets of the Influenza A virus from the HPIDB database^33^.

As for disease genes, we utilized 2,661 genes that were identified as causal for a disease as of the human phenotype ontology database (HPO)^34^ based on the Online Mendelian Inheritance in Man (OMIM) database^35^, as well as 11,002 disease genes that were identified from GWAS studies^36^.

As for drug targets, we collected a set of 2,289 drug targets that were approved by the Food and Drug Administration (FDA) as of the DrugBank database^37^ as well as 2,436 genes that were annotated as druggable^47^.

### Orthologous proteins

As a source of orthologous protein information, we utilized the OMA database that obtains ortholog groups from pairwise orthology inference and hierarchical orthologous group clustering^38^. In particular, we utilized 7,979 proteins in *M. musculus* that had orthologs to human genes in the underlying protein interaction network, 7,722 in *B. taurus*, 7,474 in *C. familiaris*, 7,697 in *G. gorilla*, 6,423 in *S. scrofa*, 5,990 in *D. rerio*, 5,874 in *G. gallus*, 5,051 in *X. tropicalis*, 1,717 in *C. elegans*, 1,427 in *A. thaliana*, 218 in *E. coli*, 2,499 in *D. melanogaster* and 893 in *S. cerevisiae*.

### Virus-host interactions

Collecting data from the HPIDB database, we obtained 988 human proteins that were targeted by the Hepatitis C virus, as well as 2,157 targets of the Herpes simplex virus, 872 targets of HIV-1 and 2,358 targets of the Influenza A virus^33^.

### Enrichment analysis

Binning proteins with a certain characteristic *d* (e.g. viral target) we calculated the fraction of proteins that had a feature *i* (e.g. bottleneck protein) in each group *d*, *f*_*i*_*(d)*. As a null model we randomly sampled protein sets with feature *i* of the same size 10,000 times and calculated the corresponding random fraction, *f*_*i*,*r*_ (*d*). The enrichment/depletion of proteins with feature *i* in a group *d* was then defined as ${E}_{i}(d)=\,l{g}_{2}({f}_{i}(d)/{f}_{i,r}(d))$. After averaging *E*_*i*_ over 10,000 randomizations, *E*_*d*_ > 0 pointed to an enrichment and *vice versa*, while *E*_*i*_ *~ 0* indicated a random process^48^.

## Supplementary information

Suppl. Material
