# Supplementary material for: Collective influencers in protein interaction networks
Source: Sci Rep. 2019 Mar 8;9:3948. doi: 10.1038/s41598-019-40410-2 (PMC6408499; doi:10.1038/s41598-019-40410-2)
Supplement: Supplementary file 1 — Suppl. Material [file 41598_2019_40410_MOESM1_ESM.docx]

**Collective influencers in protein interaction networks –**

**Suppl. Figures**

T. A. Boltz^1^, P. Devkota^1^ and Stefan Wuchty^1,2,3,4,*^

1. Department of Computer Science, University of Miami, Coral Gables, FL, USA.
2. Department of Biology, University of Miami, Coral Gables, FL, USA.
3. Sylvester Comprehensive Cancer Center, University of Miami, Miami, FL, USA.
4. Center for Computational Science, University of Miami, Coral Gables, FL, USA.

**
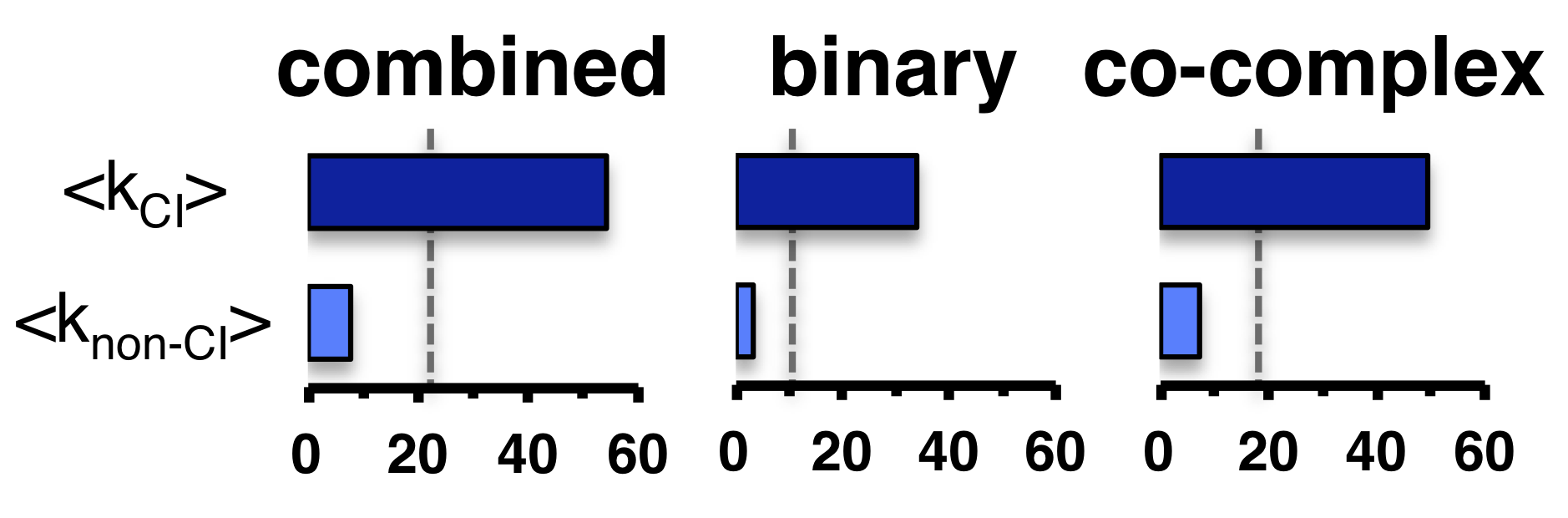
Supplementary Figure S1.** Mean Degree of (non-)CI proteins. We calculated the mean degree of (non-)CI proteins in the combined, binary and co-complex networks. We observed that CI proteins were on average involved in more interactions than non-CI proteins. Furthermore, non-CI proteins had a lower mean degree than all proteins (dashed lines).

**
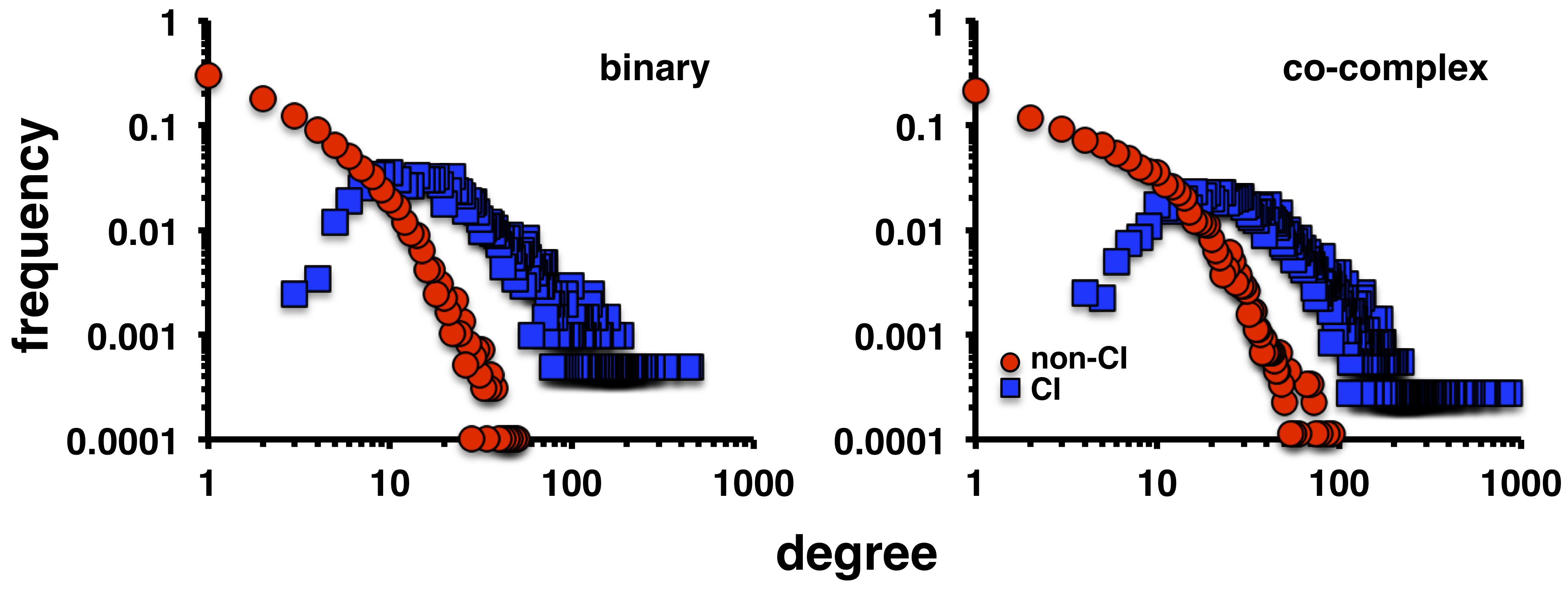
**

**Supplementary Figure S2.** Frequency distributions of degrees in the binary and co-complex network.

**
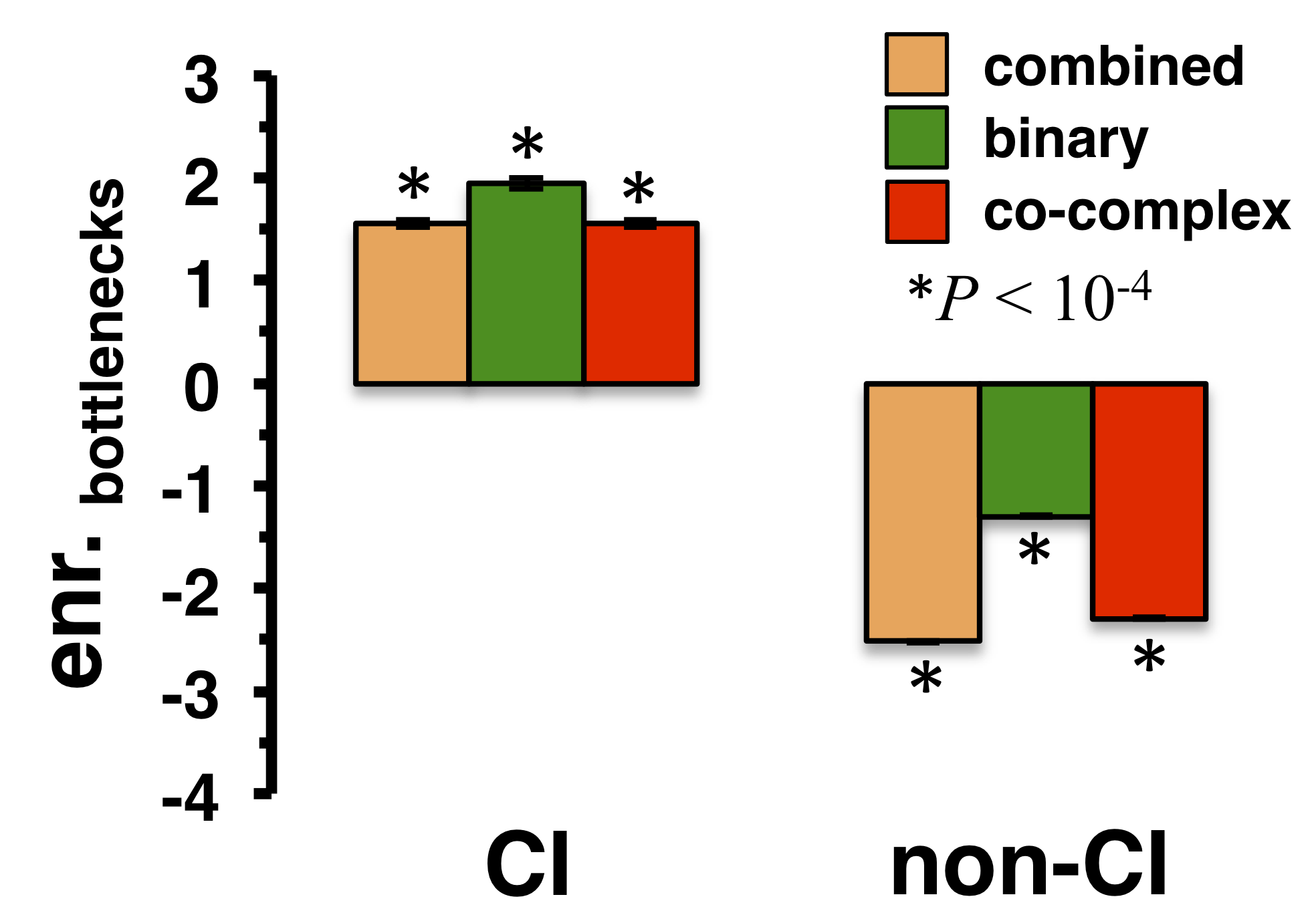
Supplementary Figure S3.** Enrichment of bottleneck nodes in sets of (non-)CI proteins. Randomizing the top 20% of proteins with highest betweeness centrality we determined the enrichment of such bottleneck proteins in the sets of (non-)CI proteins in all networks. We observed that CI proteins were significantly with bottleneck proteins.

**
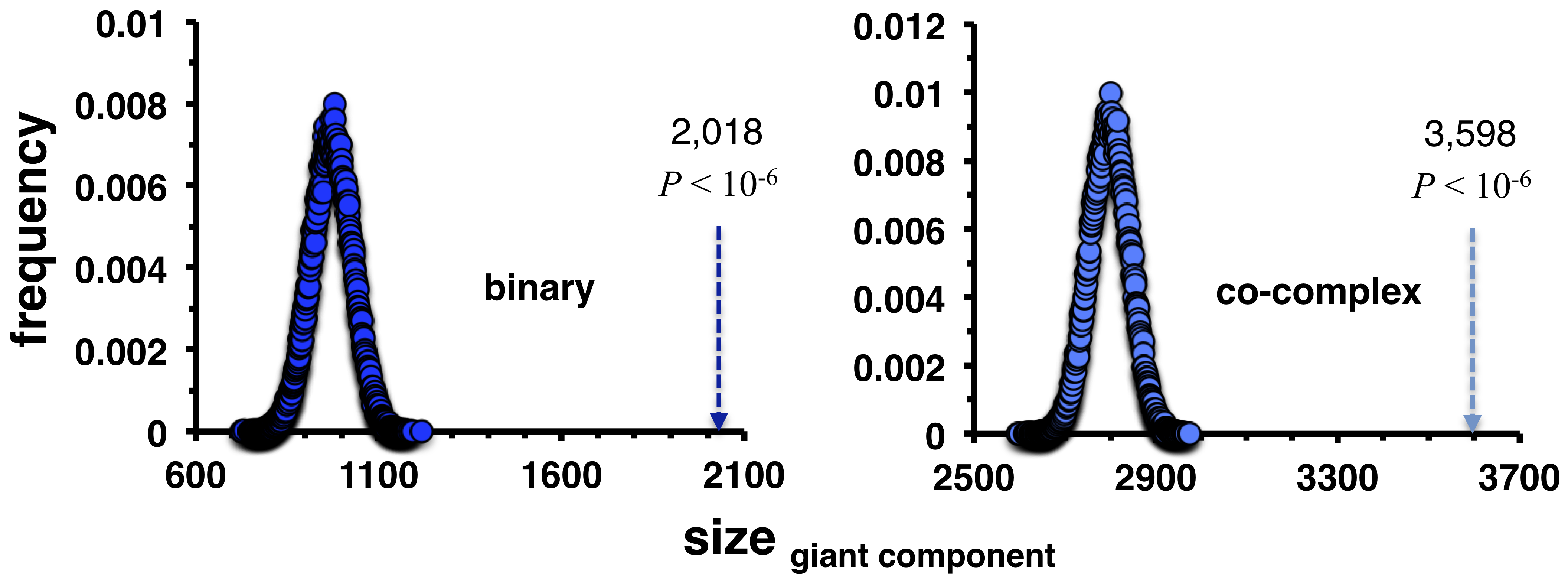
Supplementary Figure S4.** Giant connected components of CI proteins. We found that CI proteins in the binary and co-complex combined composed giant connected components with 2,018 and 3,598 CI proteins, respectively (dashed lines). Such a result was significant when we randomized sets of CI proteins and determined the distribution of the sizes of the giant components thus obtained (P < 10^-6^).

**
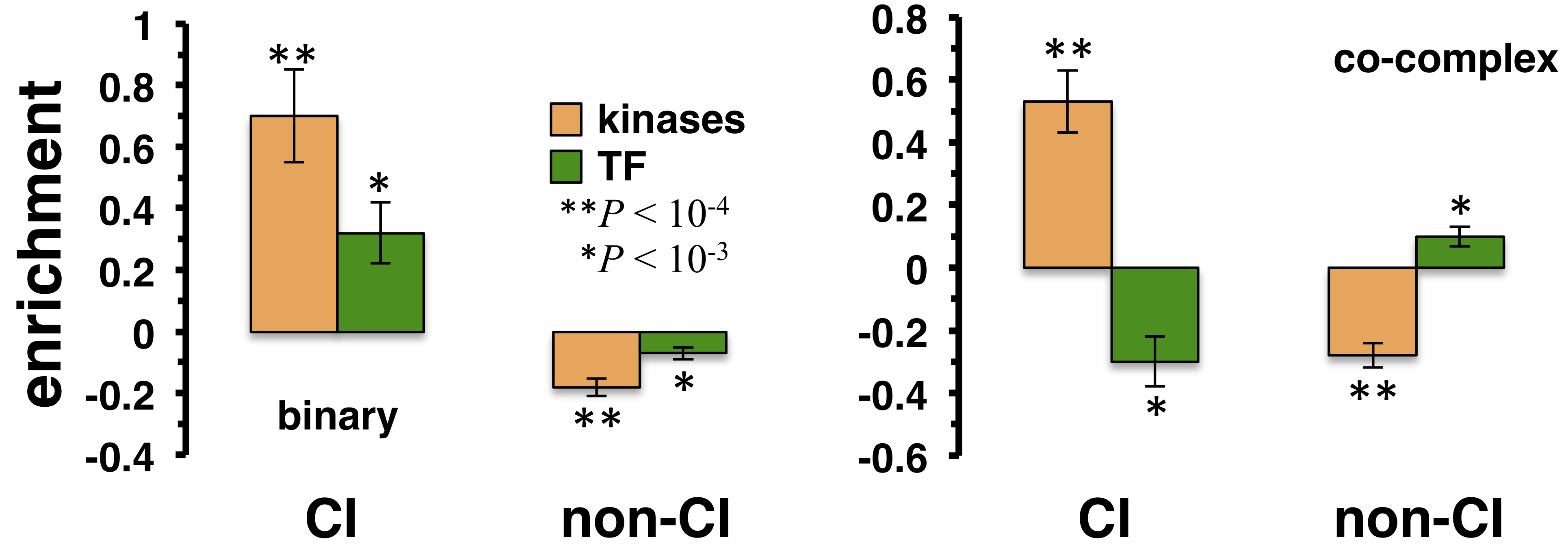
Supplementary Figure S5.** Enrichment of kinases and transcription factors genes in CIs of binary and co-complex networks. Randomizing a set of kinases we observed that such genes were significantly enriched in the sets of CIs, while we found the opposite for non-CIs in binary and co-complex networks. Notably, we found mixed results when we considered transcription factors.

**
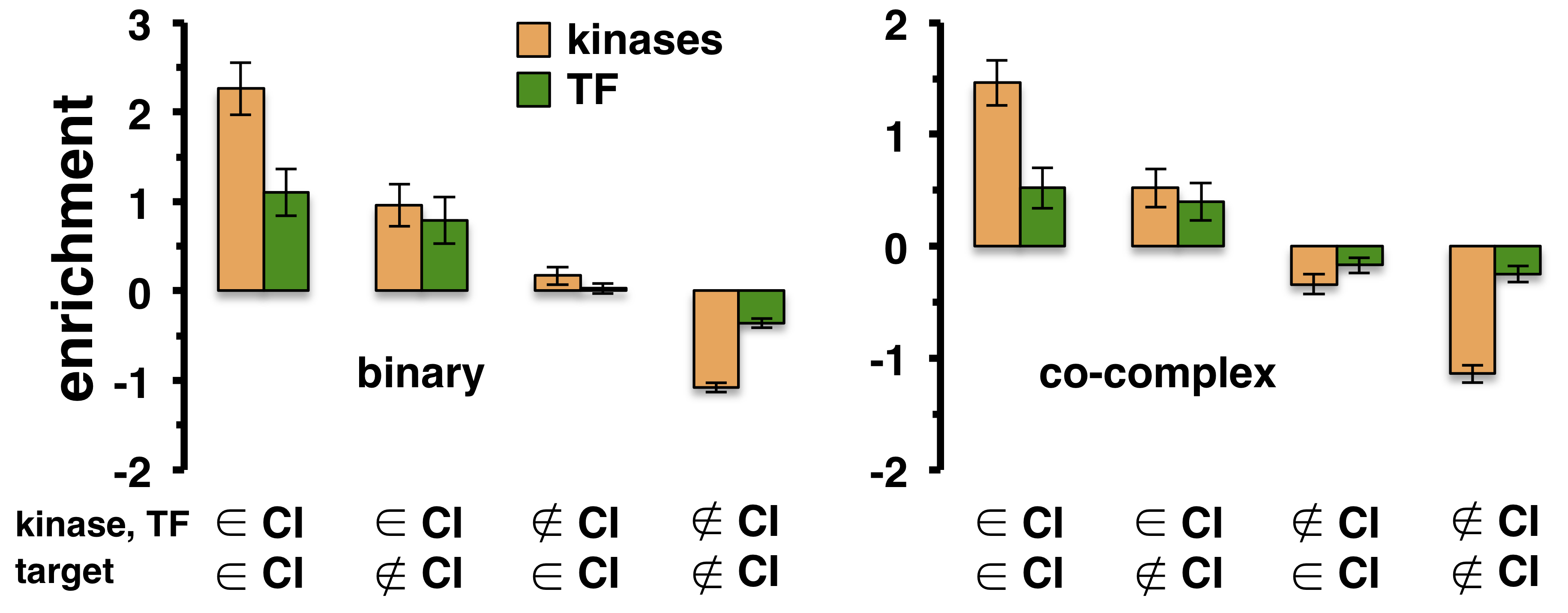
**

**Supplementary Figure S6.** Enrichment of kinases/transcription factors – target interactions in CIs of binary and co-complex networks. Randomizing sets of CI proteins in the binary and co-complex networks, we found that links between transcription factors and their corresponding targets were enriched when transcription factors were CIs. We obtained a similar result using kinase-substrate interactions.

**
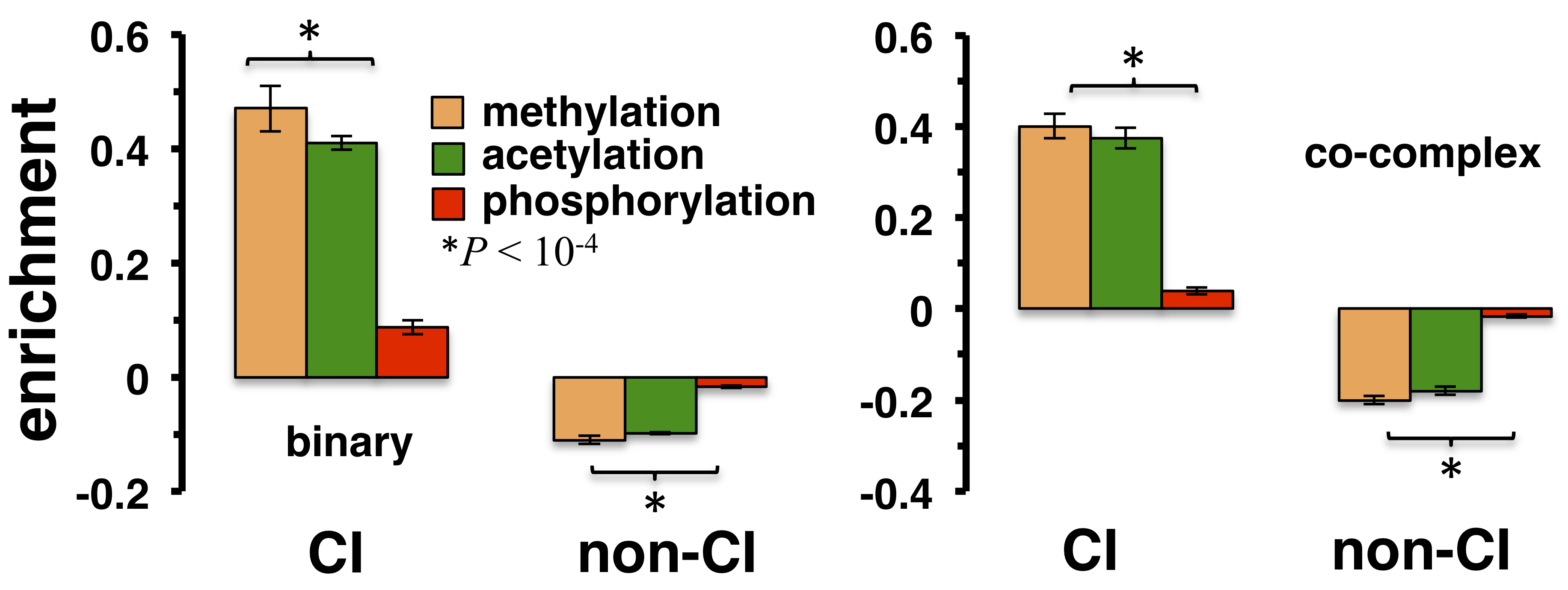
**

**Supplementary Figure S7.** Enrichment of PTM-targets with collective influencers (CI) in human protein interaction networks. As for recipients of posttranslational modifications, we observed that CIs were strongly enriched with methylation and acetylation targets in the binary and co-complex network. While still significant, CIs were less enriched with phosphorylation targets.

**
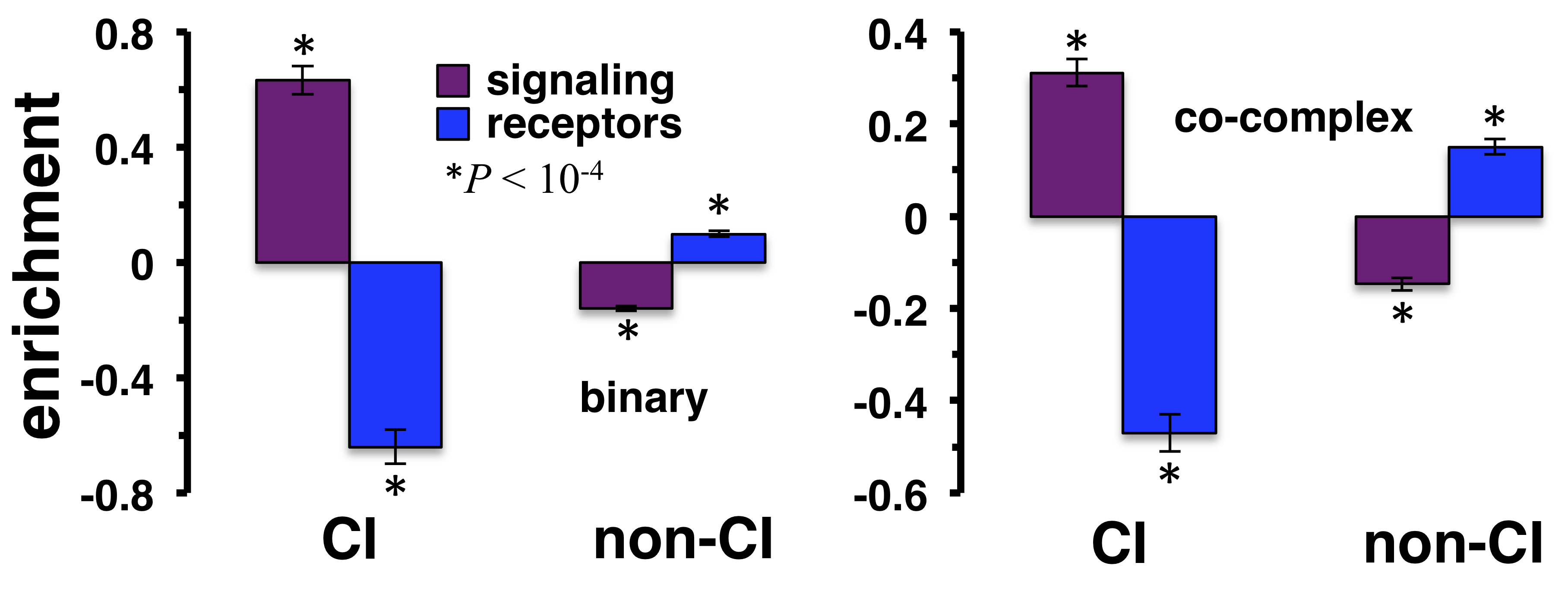
**

**Supplementary Figure S8.** Enrichment of signaling proteins with collective influencers (CI) in human protein interaction networks. While signaling proteins without membrane domains appeared strongly enriched with CIs in binary and co-complex networks we found the opposite when we considered receptors that carried a trans-membrane protein in the combined network.

**
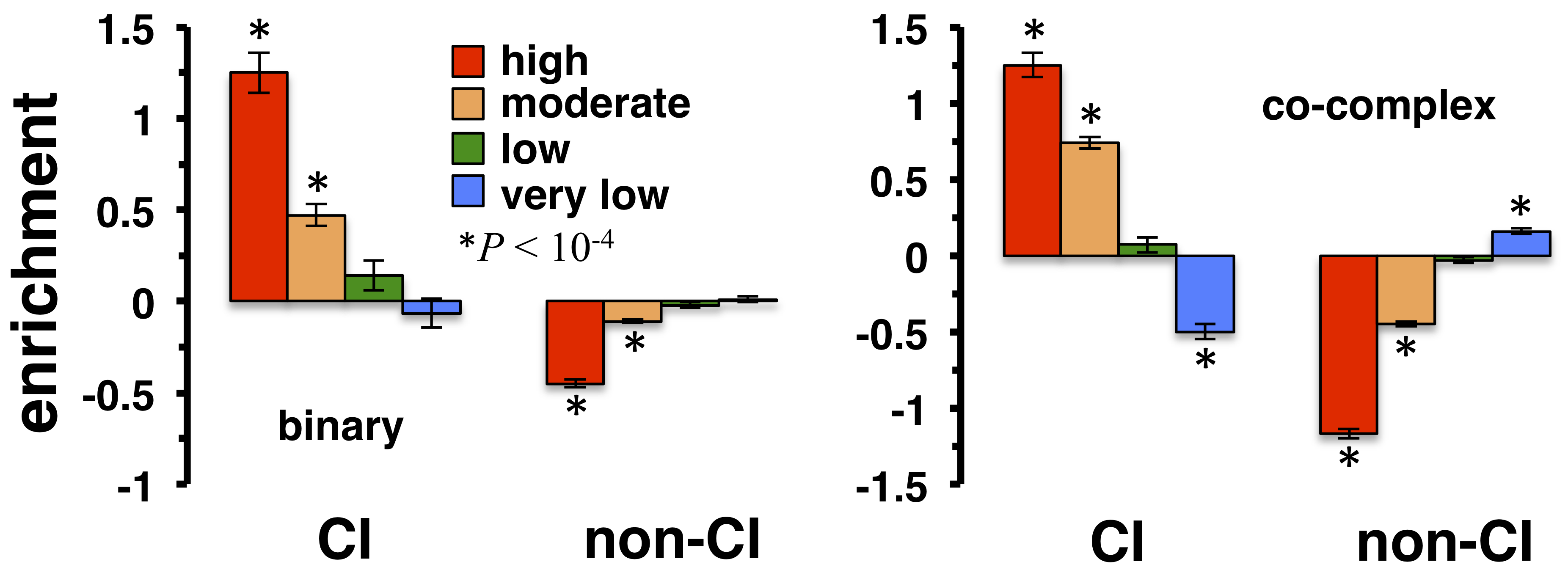
**

**Supplementary Figure S9.** Enrichment of abundant proteins with collective influencers (CI) in human protein interaction networks. As a measure of translational regulation, we found that CIs in the binary and co-complex networks were significantly enriched with high copy number proteins, while they were depleted with low copy number proteins.

**Supp
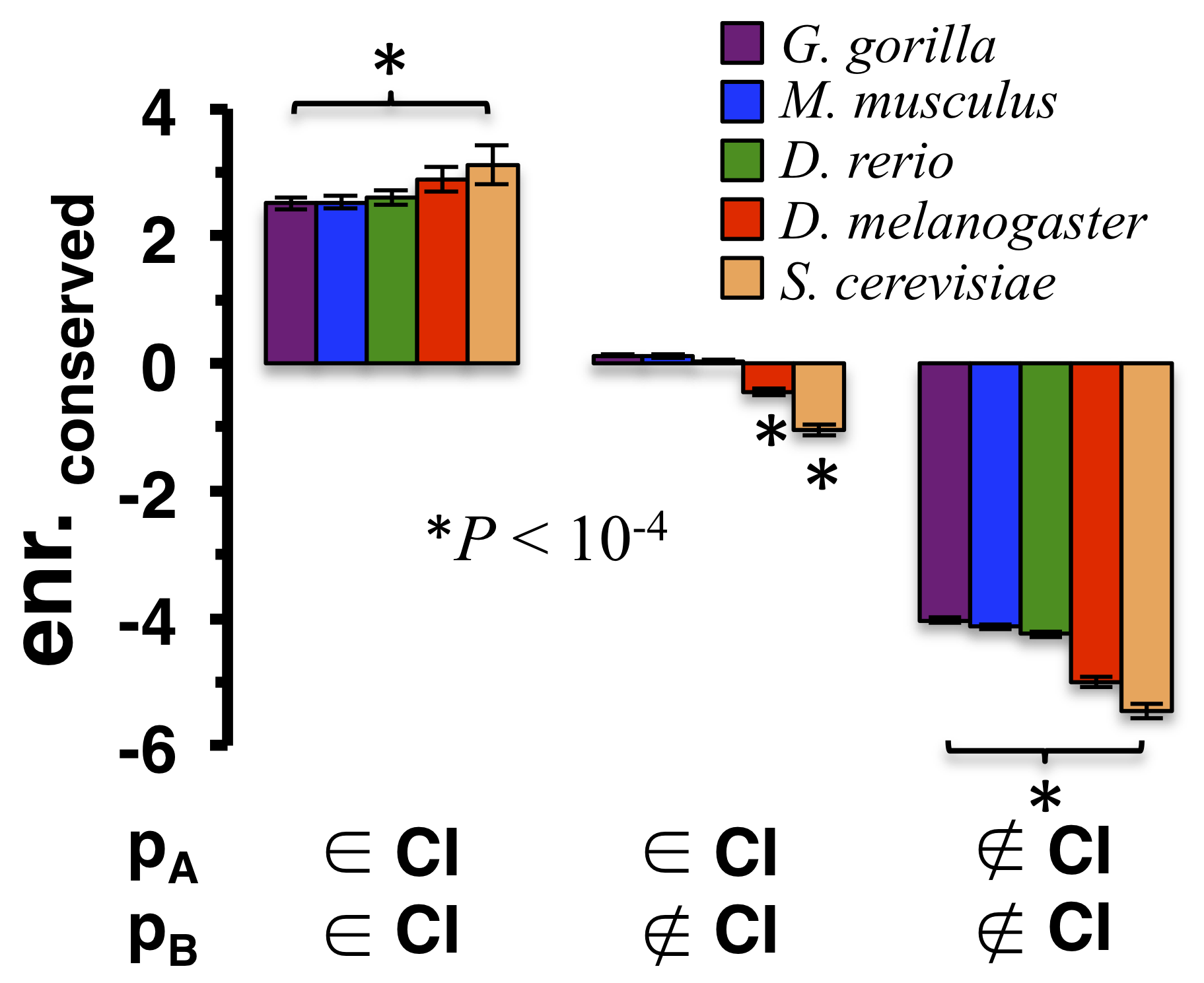
lementary Figure S10.** Enrichment of interactions between conserved CI proteins. Randomizing sets of human proteins that have orthologs in different organisms, we determined the enrichment of interactions between conserved (non-)CI proteins in the combined network. We found that interactions between CI proteins were preferably enriched with orthologs while interactions between non-CI proteins were depleted.
